# PRIMO Monte Carlo software benchmarked against a reference dosimetry dataset for 6 MV photon beams from Varian linacs

**DOI:** 10.1186/s13014-018-1076-0

**Published:** 2018-08-07

**Authors:** Marcelino Hermida–López, David Sánchez–Artuñedo, Juan Francisco Calvo–Ortega

**Affiliations:** 1 Servei de Física i Protecció Radiològica. Hospital Universitari Vall d’Hebron, Pg. Vall d’Hebron, 119–129, Barcelona, 08035 Spain; 2 Servicio de Oncología Radioterápica, Hospital Quirónsalud Barcelona, Pza. Alfonso Comín, 5, Barcelona, 08023 Spain; 3 Servicio de Oncología Radioterápica, Hospital Universitario Dexeus, C./ Sabino Arana, 5-19, Barcelona, 08028 Spain

**Keywords:** PRIMO, Monte Carlo simulation, Dosimetry, Linac

## Abstract

**Background:**

The software PRIMO for the Monte Carlo simulation of radiotherapy linacs could potentially act as a independent calculation system to verify the calculations of treatment planning systems. We investigated the suitability of the PRIMO default beam parameters to produce accurate dosimetric results for 6 MV photon beams from Varian Clinac 2100 linacs and 6 MV flattening–filter–free photon beams from Varian TrueBeam linacs.

**Methods:**

Simulation results with the DPM algorithm were benchmarked against a published reference dosimetry dataset based on point measurements of 25 dosimetric parameters on a large series of linacs. Studied parameters (for several field sizes and depths) were: PDD, off–axis ratios, and output factors for open fields and IMRT/SBRT–style fields. For the latter, the output factors were also determined with radiochromic film and with a small–sized ionization chamber. Benchmark data, PRIMO simulation results and our experimental results were compared.

**Results:**

PDD, off–axis ratios, and open–field output factors obtained from the simulations with the PRIMO default beam parameters agreed with the benchmark data within 2.4% for Clinac 2100, and within 1.3% for TrueBeam. Higher differences were found for IMRT/SBRT–style output factors: up to 2.8% for Clinac 2100, and up to 3.3% for TrueBeam. Experimental output factors agreed with benchmark data within 1.0% (ionization chamber) and within 1.9% (radiochromic film).

**Conclusions:**

PRIMO default initial beam parameters for 6 MV photon beams from Varian Clinac 2100 linacs and 6 MV FFF photon beams from Varian TrueBeam linacs allowed agreement within 3.3% with a dosimetry database based on measurements of a high number of linacs. This finding represents a first step in the validation of PRIMO for the independent verification of radiotherapy plans.

**Electronic supplementary material:**

The online version of this article (10.1186/s13014-018-1076-0) contains supplementary material, which is available to authorized users.

## Background

The Monte Carlo simulation of radiation transport is considered the gold standard method in radiation transport calculations, and has been successfully applied to the simulation of radiotherapy linacs since the 1980s [1]. Major drawbacks have been the long computation times not suitable for the routine clinical practice, and the effort needed to develop the simulation of a radiotherapy linac from scratch.

The software PRIMO [2, 3] was introduced to overcome such limitations. PRIMO performs the Monte Carlo simulation of radiotherapy linacs in an user–friendly manner, estimating absorbed dose distributions in slab phantoms of arbitrary composition, and in computed tomography (CT) sets. It can be freely downloaded from https://www.primoproject.net/. PRIMO supports two simulation engines: the general–purpose Monte Carlo code PENELOPE 2011 [4] combined with the steering program PENEASY [5], and the Dose Planning Method (DPM) [6], which is a Monte Carlo algorithm optimized for the simulation of electron–photon showers under radiotherapy conditions.

PRIMO characterizes the initial electron beam with the following user–editable parameters: mean energy, energy full–width at half–maximum (FWHM), focal spot FWHM, and beam divergence. With an adequate tuning of these parameters, a good agreement can be achieved between PRIMO simulation results and measurements [7]. To reduce the time needed for this tuning process, the software suggests default values of the initial beam parameters for each nominal energy of the available linac models.

In the latest available version, PRIMO introduced the capability of simulating clinical intensity–modulated radiation therapy plans (IMRT) and volumetric modulated arc therapy (VMAT) plans, from Varian linacs (Varian Medical Systems, Palo Alto, CA, USA). Hence, PRIMO could potentially perform independent calculations to verify the calculations of treatment planning systems (TPS). To that purpose, a comprehensive dosimetric validation of PRIMO would be necessary. This work is a first step in such a validation.

The TG–114 report of the American Association of Physicists in Medicine (AAPM) [8] gives two general requirements to achieve a truly independent calculation system: it should be based on a different algorithm from the TPS, and the beam data should also be different from those used by the TPS. PRIMO fulfills the first requirement, as the implemented Monte Carlo algorithms are not used by any TPS. We used the PRIMO defaults for the initial beam parameters to comply with the second requirement, instead of tuning PRIMO to match a specific linac. If we tune the PRIMO simulation parameters to match the simulation results to beam data from a particular linac, a possible flaw in the measured data will also propagate to the simulation results. A possible solution is to use simulation parameters that reproduce dose distributions representative of the linac model, rather than a particular linac. The present work aims to prove that the default simulation parameters produce such dose distributions.

We investigated the suitability of the PRIMO default beam parameters to produce accurate dosimetric results, by comparing dosimetric parameters from PRIMO simulations using the DPM algorithm against a published dataset based on measurements on large series of linacs of the same model. We focused on 6 MV photon beams from Varian Clinac 2100 linacs and on 6 MV flattening–filter free (FFF) photon beams from Varian TrueBeam linacs, both with a Millennium 120 multileaf collimator (MLC).

## Methods

A published dataset of experimental dosimetric parameters was used to benchmark the simulation results. For the most dosimetrically challenging parameters (small–field output factors), apart from the simulations, we carried out experimental measurements with radiochromic film and with a small–sized ionization chamber, for the 6 MV photon beam from a Clinac 2100 CD linac. The benchmark data, the PRIMO simulation results, and our experimental results were compared.

### Benchmark dosimetry dataset

The Imaging and Radiation Oncology Core–Houston (IROC-H) Quality Assurance Center (formerly named the Radiological Physics Center) was established in 1968 to ensure the quality of radiotherapy treatments of institutions participating in clinical trials. Among other services, IROC–H performs on–site dosimetry audits, which involve the acquisition of basic dosimetric parameters of the audited linacs. Through these audits, IROC–H has compiled the most comprehensive dosimetry dataset of radiotherapy linacs available to date [9–11], including approximately 500 Varian machines. The data were obtained by IROC–H staff physicists following consistent standard procedures including a check by a second physicist. These dosimetric data were classified by beam energy, and similarly performing machines were clustered into different classes.

The IROC–H reference dosimetry dataset reported by Kerns et al. [11] was used in this work to benchmark the results of the PRIMO simulations. Table 1 summarizes the dosimetric parameters reported and the number of Clinac 2100 and TrueBeam linacs studied. The parameters were determined by IROC–H measuring at the point locations specified at Table 1, and are the following: percentage depth–doses (PDD), off–axis ratios (only for a 40×40 cm^2^ field size), open–field (i.e., with the MLC retracted) output factors (OF) at the depth of the maximum dose (*d*_*max*_), and OF for *IMRT–style* and *SBRT–style* fields, both at a depth of 10 cm. In IMRT–style fields, the jaws were fixed at 10×10 cm^2^ and the effective field size was defined by the MLC, while in SBRT–style fields both jaws and MLC moved to define the field size. These fields try to approximate typical segments of a IMRT field, and jaw positions of a SBRT field.

**Table 1:** Dosimetric parameters reported by IROC–H [11] for 6 MV beams from Clinac 2100 linacs, and for 6 MV FFF beams from TrueBeam linacs

Parameter	Field size (cm^2^)	*N* Clinac 2100	*N* TrueBeam
PDD at *d*_*max*_	10 ×10	116	11
PDD at 5, 15, and 20 cm	6 ×6	99	11
	10 ×10	116	11
	20 ×20	99	11
Off-axis ratios at 5 cm left,	40 ×40	116	11
10 cm avg.,			
and 15 cm left			
Open field OF at *d*_*max*_	6 ×6	104	11
	15 ×15		
	20 ×20		
	30 ×30		
IMRT-style OF at 10 cm	2 ×2	4	11
	3 ×3		
	4 ×4		
	6 ×6		
SBRT-style OF at 10 cm	2 ×2	20	4
	3 ×3		
	4 ×4		
	6 ×6		

*N* represents the number of linacs studied for each parameter. Data for Clinac 2100 were taken from the class ‘2100’, except for the SBRT–style OF, which were taken from the class ‘Base’. Data for TrueBeam were taken from the class ‘TB–FFF’

IROC–H measurements were performed with a 30×30×30 cm^3^ water phantom placed at a source-to-surface distance of 100 cm. A calibrated Exradin A12 Farmer–type chamber (Standard Imaging Inc., Madison, WI, USA) was used, except for the SBRT– and IMRT–style fields, for which the chosen detector was a Exradin A16 microchamber, with a sensitive volume of 0.007 cm^3^. It is worth mentioning that to determine the off–axis ratios of the 40×40 cm^2^ field, the chamber was placed in the middle of the phantom, and then the phantom was shifted laterally to each measurement location (S. F. Kry, personal communication, Jan. 21, 2018).

The benchmark data for the Clinac 2100 were taken from the class ‘2100’, except for the SBRT–style OF, which were taken from the class ‘Base’. Data for TrueBeam were taken from the class ‘TB–FFF’. The median values reported by IROC–H for each parameter listed at Table 1 were used to compare with our simulation results and measurements.

### PRIMO simulations

We used PRIMO to calculate the dosimetric parameters described in Table 1, reproducing as close as possible the experimental setups used by IROC–H. Table 2 details the simulation conditions used in this work. The table follows the template proposed by the report RECORDS [12] from the Task Group 268 of the AAPM.

**Table 2:** Simulation conditions used in this work, displayed as per the scheme proposed by the RECORDS report [12]

Item	Description	References
Code	PRIMO v. 0.3.1.1600, based on penEasy/penelope 2011, and DPM algorithm	[2–6]
Timing PSF	Simulation time: ≈10 d on an Intel Xeon E5-2670 v3, 24 cores @ 2.3 GHz, 64 GB RAM, Windows Server 2016	
Timing 10×10 cm^2^ field	Simulation time with DPM: 5.5 h on an Intel Xeon E5-2620. 2 CPU (×6 cores) @ 2.00 GHz, 32 GB RAM, Windows 7. Other fields: CPU time linearly proportional to the field area.	
Source description Clinac 2100	PSF stage 1 simulated with PRIMO, 6 MV, initial energy: 5.4 MeV, energy FWHM: 0, focal spot FWHM: 0, beam divergence: 0, field size: 40 ×40 cm^2^. Simulation engine: penEasy/penelope. Histories: 850 ×10^6^. PSF size: 150 GB	
Source description TrueBeam	PSF stage 1 simulated with PRIMO, FakeBeam, 6 MV FFF, initial energy: 5.8 MeV, energy FWHM: 0.058 MeV, focal spot FWHM: 0.15 cm, beam divergence: 0, field size: 40 ×40 cm^2^. Simulation engine: penEasy/ penelope. Histories: 850 ×10^6^. PSF size: 237 GB	
Cross sections	penelope 2011 and DPM.	[4, 6]
Transport parameters	PRIMO default transport parameters for 6 MV from Clinac 2100 and for 6 MV FFF from FakeBeam	[20]
Variance-reduction techniques	PSF simulations: splitting roulette. Movable–skins technique applied to the simulation of primary collimator, jaws and MLC. Water phantom simulations: particle splitting (×170, factor empirically determined)	[20, 23, 24]
Scored quantities	Absorbed dose to a voxelized water phantom of 30.2 ×30.2×30 cm^3^, voxel size 0.2 ×0.2×0.2 cm^3^. Simulation engine: DPM and penEasy/ penelope 2011	
# histories/ statistical uncertainty	850 ×10^6^ histories. Statistical uncertainty of the calculated dosimetric parameters typically below 2% (*k*=2), estimated with the history–by–history method of penelope 2011	[4, 20]
Post-processing	No smoothing or de–noising was applied to the simulation results.	

We used the latest released version of PRIMO (v. 0.3.1, Jan. 2018). PRIMO simulates most Varian linacs, with several MLC models. PRIMO includes the geometries of the supported linacs which were coded from blueprints provided by the manufacturers. The only exception is the Varian TrueBeam linac, which is simulated using an approximate empirical geometry named FakeBeam, developed by the PRIMO authors [7].

PRIMO allows dividing the full simulation of the linac and phantom/CT set in three separate stages: the first stage (*s1*) simulates the upper, field–independent, part of the linac, that is, from the exit of the accelerating waveguide to just above the jaws. The second stage (*s2*) simulates the lower, field–dependent, part of the linac (jaws and MLC). Finally, the simulation of the dose deposition in a slab phantom or in a CT set is named *s3* stage.

First, using PENEASY/PENELOPE as simulation engine, we obtained phase-space files (PSF) of the *s1* stage for each of the studied configurations: Clinac 2100 with a 6 MV photon beam and a FakeBeam with a 6 MV FFF photon beam. In both cases, the default initial beam parameters suggested by PRIMO were used (see Table 2).

The PSFs obtained in simulations of stage *s1* were used as the source of particles for the joined simulation of the *s2* and *s3* stages for the fields specified in Table 1.

DPM was the simulation engine used in *s2* and *s3* stages. DPM is the preferred choice for independent calculation of clinical plans due to its higher performance compared to PENELOPE.

Absorbed dose was tallied in a voxelized water phantom. The beam axis was located at the center of the phantom surface. The phantom was positioned and binned such that all the measurement locations coincided with the coordinates of the center of a bin. Therefore, no interpolation was performed to sample the dose from the bins used in calculations. The uncertainties of the calculated dosimetric parameters were obtained from the statistical uncertainties of the simulated doses, by applying the usual rules of uncertainty propagation.

### Radiochromic film measurements

Measurements of the OF for the IMRT– and SBRT–style fields (Table 1) were carried out using EBT3 radiochromic film (Ashland Inc., Wayne, NJ, USA). Film was used according to recommendations from the manufacturer and the literature [13], and to our previous experience [14].

All film pieces were obtained from the same lot (# 05011703). As the response of the radiochromic film is sensitive to the film orientation on the scanner bed, all the film pieces were scanned in the same orientation that was used with the calibration films (portrait). All irradiations were done with a 6 MV photon beam from a Clinac 2100 CD linac equipped with a Millennium 120 MLC.

Three measurement sessions at different dates were performed. On every session, each IMRT/SBRT field was delivered onto a 5×5 cm^2^ film piece placed at a depth of 10 cm in a MP3 water phantom (PTW, Freiburg, Germany), with a source–to–surface distance of 100 cm. The dose received by the films for each IMRT/SBRT field was around 1.2 Gy. Film pieces were supported with the holder of a plane–parallel ionization chamber. Before immersion in water, the linac cross–hair projection was marked on each film. Each IMRT/SBRT field was delivered three times over three film pieces (one irradiation per piece). In addition, for each measurement session, one 5×5 cm^2^ film piece was exposed to the 10×10 cm^2^ reference field.

After exposures, the films were dried, and stored in a dark place. Twenty–four hours after irradiation, the four pieces corresponding to each IMRT/SBRT case, alongside a 20×4 cm^2^ unexposed piece, were simultaneously scanned at the central region of a flatbed scanner Epson Perfection V750 Pro (Seiko Epson Corporation, Nagano, Japan). RGB positive images were taken at a color depth of 16 bits per color channel, with a resolution of 72 dpi, and with the image processing tools turned off. A 1 mm-thick glass sheet was placed over the films to avoid film curling and the Callier effect [15].

The images were uploaded into the web–based application for film dosimetry https://www.radiochromic.com(v. 3.0), which introduces a novel multichannel algorithm to improve dose accuracy [16]. A calibration curve (pixel value–dose) from 0 to 5 Gy was established for each measurement session. The unexposed pieces are used in https://www.radiochromic.com to account for inter–scan variations [17]. For each IMRT/SBRT case, doses at the central point indicated by a cross-hair passing through the four marks showing the linac cross-hair was obtained for the four exposed films. Then, the OF value for each IMRT/SBRT case was calculated as the ratio of the dose of the IMRT/SBRT field to the dose of the 10×10 cm^2^ reference field of each measurement session. The average OF values resulting from this procedure are reported. The Additional file 1 supply images on the experimental setup and the film reading.

### Ionization chamber measurements

For comparison with the EBT3 results, the OF of the IMRT– and SBRT–style fields were also determined in the IROC–H conditions for the 6 MV photon beam of the same Clinac 2100 CD linac. A PTW 31014 PinPoint chamber in conjunction with a PTW Unidos electrometer (PTW, Freiburg, Germany) were used. The PinPoint chamber has a sensitive volume of 0.015 cm^3^.

The chamber was set up in the PTW MP3 water phantom with its axis coincident with the beam axis. The positioning of the chamber at the radiation beam center was checked by acquiring cross– and in–plane radiation profiles. OF measurements were performed in two sessions on different dates, and the average OF values are reported.

For small fields, to obtain the correct OF from the ratio of readings provided by a ionization chamber, it is necessary to apply a OF correction factor to convert the ratio of ionization readings to a true dose ratio [18]. Such OF correction factors depend on the detector used, and become greater as the field size becomes smaller. The TRS–483 report on small–field dosimetry [19] collects such OF correction factors for commonly used detectors. The appropriate OF correction factors were applied to the PinPoint chamber readings.

For completeness, apart from the OF for IMRT– and SBRT–style fields, we also determined the rest of the parameters from Table 1 (using a PTW 31002 Semiflex ionization chamber, with a sensitive volume of 0.125 cm^3^).

### Experimental uncertainties

The experimental uncertainties of the OF determined with the EBT3 film and the PinPoint chamber were estimated by assessing the following sources (uncertainties reported with *k*=2):

- EBT3 film: i) fitting procedure of the pixel value–dose calibration curve (2%); ii) repeatability of the scanner response (1.0%); iii) intra–lot film reproducibility (1.8%); iv) film noise (1.0%); and v) linac output repeatability (0.4%). The lateral scanner effect [13] was not considered as the films were always placed at the center of the scanner bed. The overall uncertainty in the OF values resulted of 4.2%.
- PinPoint chamber: i) chamber setup (1.0%); ii) reading correction for influence quantities as pressure, temperature, polarity and recombination (0.8%); iii) uncertainty of the OF correction factor (0.8%) [19]; and v) linac output repeatability (0.4%). An overall uncertainty in the measured OF of 1.6% was obtained.

## Results

The mean and maximum statistical uncertainties (with *k*=2) of the simulated parameters for Clinac 2100 were 1.6% and 1.8%, respectively. For TrueBeam, those values were 0.7% and 1.5%, respectively.

**Fig. 1:**
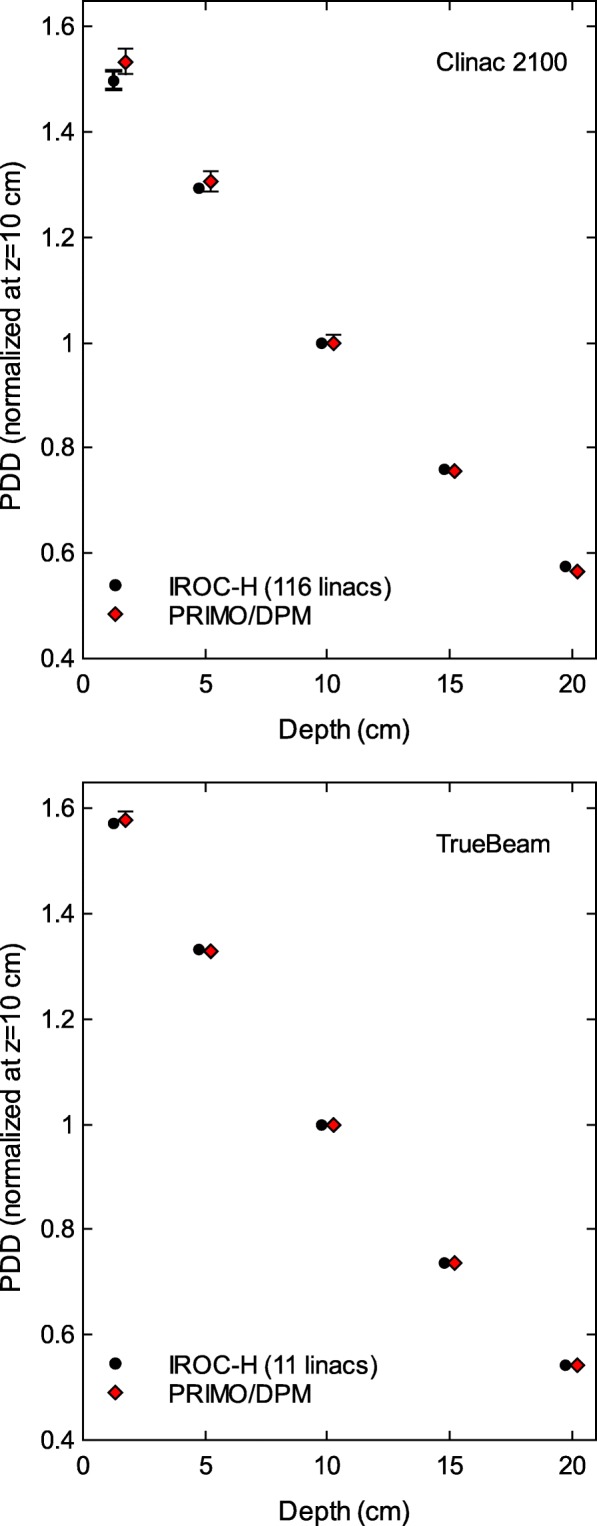
Percentage depth–doses of a 10×10 cm^2^ field reported by IROC–H, and calculated with PRIMO using the DPM algorithm. The maximum differences between simulations and IROC–H data are 2.3*%* for Clinac 2100, and 0.6*%* for TrueBeam. Uncertainty bars show (with *k*=2) the standard deviation of the IROC–H data, and the statistical uncertainty of the simulations. For most data points, the bars are smaller than the symbol size. Data points are artificially separated along the horizontal axis for clarity

Figure 1 shows the comparison between the IROC–H data and the simulation results for the PDD of the 10×10 cm^2^ field, both for the Clinac 2100 and TrueBeam linacs. The maximum difference for the Clinac 2100 PDD is 2.3%, whereas the maximum difference is below 0.6% for the TrueBeam PDD. A similar trend was found for the PDD of the 6×6 cm^2^ and 20×20 cm^2^ fields, with maximum differences of − 2.4*%* for Clinac 2100, and of 0.3*%* for TrueBeam.

**Fig. 2:**
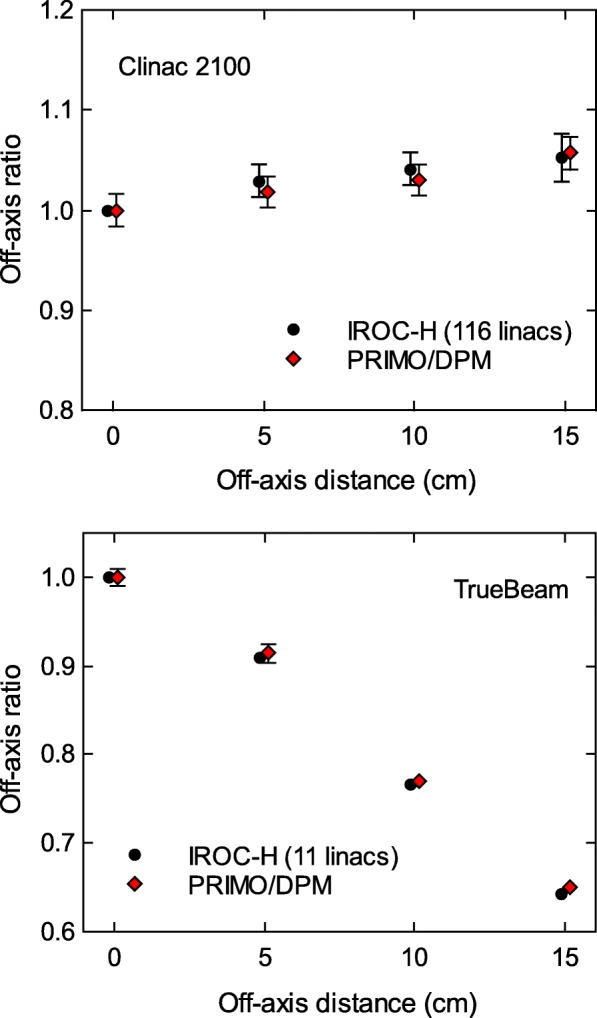
Off–axis ratios for a 40×40 cm^2^ field reported by IROC–H, and calculated with PRIMO using the DPM algorithm. The maximum differences between simulations and IROC–H data are − 1.1*%* for Clinac 2100, and 1.3*%* for TrueBeam. Uncertainty bars show (with *k*=2) the standard deviation of the IROC–H data, and the statistical uncertainty of the simulations. For some data points, the bars are smaller than the symbol size. Data points are artificially separated along the horizontal axis for clarity

Figure 2 shows the comparison between the IROC–H data and the simulation results for the off–axis ratios of the 40×40 cm^2^ field, both for the Clinac 2100 and TrueBeam linacs. The measured data and the simulation results agree within the experimental and statistical uncertainties, with maximum differences of − 1.1*%* for the Clinac 2100, and of 1.3*%* for the TrueBeam.

**Fig. 3:**
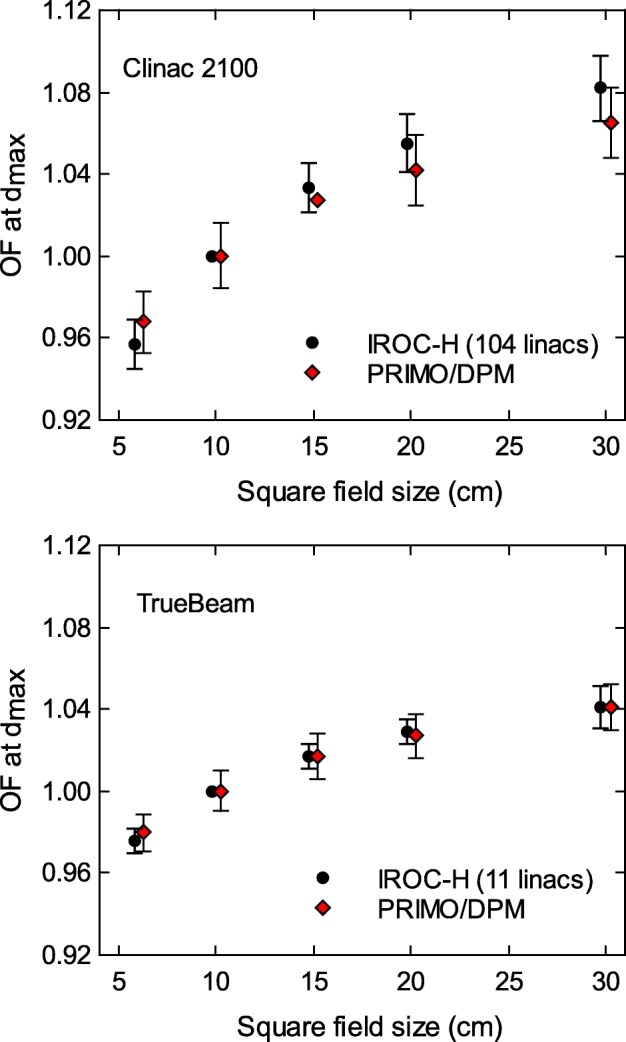
Output factors for open fields at *d*_*max*_ reported by IROC–H, and calculated with PRIMO using the DPM algorithm. The maximum differences between simulations and IROC–H data are − 1.6*%* for Clinac 2100, and 0.4*%* for TrueBeam. Uncertainty bars show (with *k*=2) the standard deviation of the IROC–H data, and the statistical uncertainty of the simulations. Data points are artificially separated along the horizontal axis for clarity

Figure 3 shows the comparison between the IROC–H data and the simulation results for the open-field OF at *d*_*max*_, both for the Clinac 2100 and TrueBeam linacs. Experimental and simulated OF agree within the uncertainties, although the maximum difference is appreciably lower for TrueBeam (0.4*%*) than for the Clinac 2100 (− 1.6*%*).

**Fig. 4:**
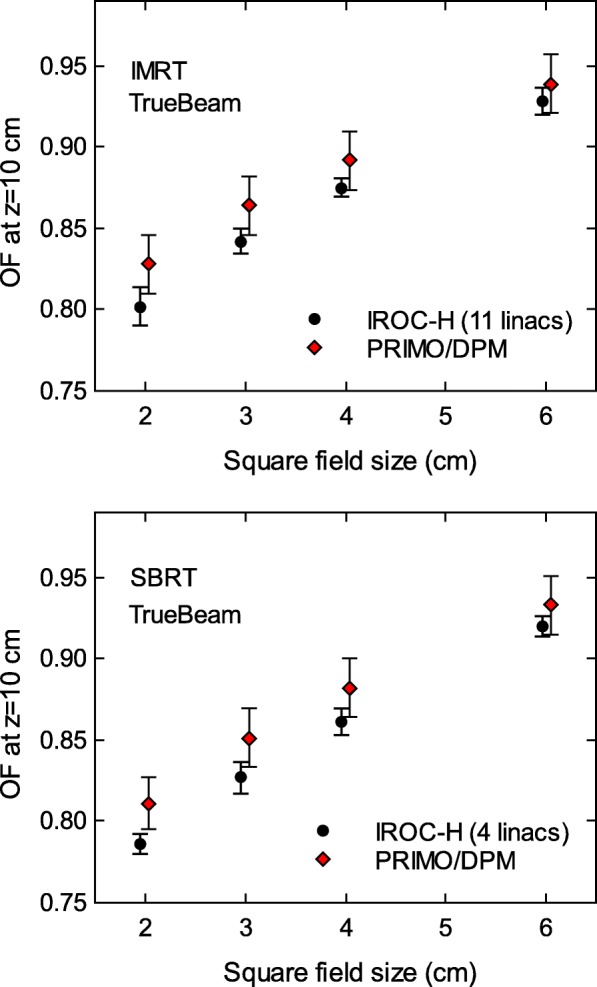
Output factors at a depth of 10 cm for IMRT– and SBRT–style fields from TrueBeam, as reported by IROC–H, and calculated with PRIMO using the DPM algorithm. The maximum differences of each field type between simulations and IROC–H data are 3.3*%* for IMRT 2×2 cm^2^ field, and 3.2*%* for SBRT 2×2 cm^2^ field. Uncertainty bars show (with *k*=2) the standard deviation of the IROC–H data, and the statistical uncertainty of the simulations. Data points are artificially separated along the horizontal axis for clarity

**Fig. 5:**
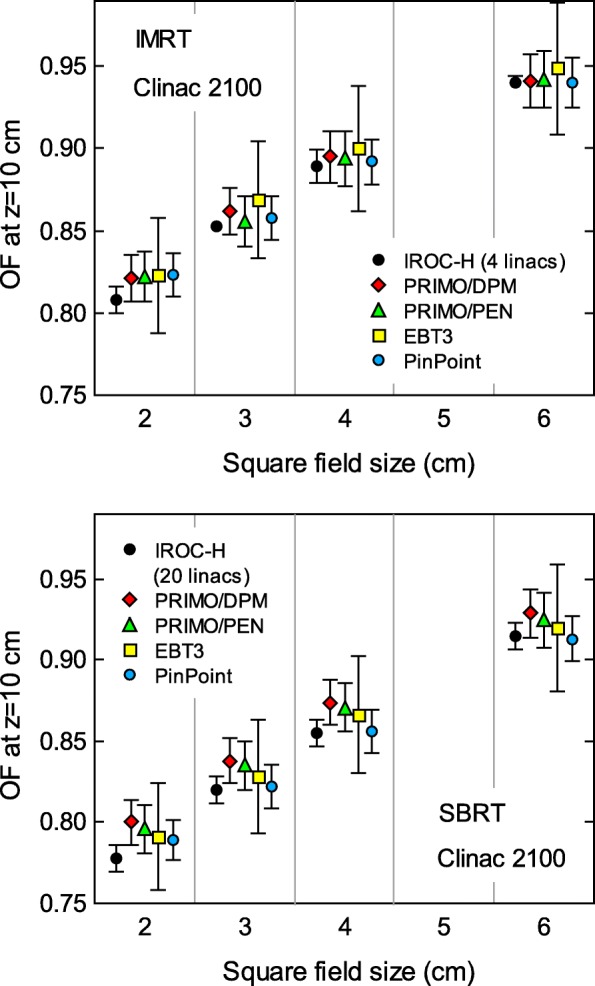
Output factors for IMRT– and SBRT–style fields from Clinac 2100, with sizes of 2×2 cm^2^, 3×3 cm^2^, 4×4 cm^2^, and 6×6 cm^2^. The graphs show the IROC–H data, results from simulations with PRIMO/DPM and PRIMO/PENELOPE, and measurements from this work with EBT3 film and a PinPoint 31014 chamber. Uncertainty bars show (with *k*=2) the standard deviation of the IROC–H data, the statistical uncertainty of the simulations, and the estimated experimental uncertainty of the measurements. For some data points, the bars are smaller than the symbol size. Data points for each field size are artificially separated along the horizontal axis for clarity

Bigger differences between IROC–H OF data and simulations arose for the IMRT– and SBRT–style fields. Figure 4 shows the results for the TrueBeam, with differences between 1.2*%* and 3.3*%* for the IMRT–style fields, and between 1.4*%* and 3.2*%* for the SBRT–style fields. The agreement is better for Clinac 2100 (Fig. 5), with differences between 0.2*%* and 1.6*%* for the IMRT–style fields, and between 1.6*%* and 2.8*%* for the SBRT–style fields.

Figure 5 also shows the OF for IMRT– and SBRT–style fields obtained from the EBT3 film and PinPoint chamber measurements. Our PinPoint experimental data and IROC–H data agree within 1.0%. For the rest of the dosimetric parameters from Table 1 (determined with the Semiflex chamber), the agreement is within 0.4% (not shown). Thus, the Clinac 2100 CD used in this work is a ‘typical’ Clinac 2100 linac, according to IROC–H data. The OF determined with the EBT3 film agree within 1.9% with the IROC–H data.

The OF obtained with PinPoint and EBT3 agree within 1.4%, a value smaller than the experimental uncertainties (1.6% for PinPoint OF, and 4.2% for EBT3 OF). A good agreement between OF determined with detectors based on different physical principles gives confidence on the accuracy of the results [18].

Tables showing a comparison between the parameters obtained from the simulations, the measurements and the IROC–H data, can be found in the Additional file 1.

## Discussion

PDD, OF at *d*_*max*_, and off–axis ratios obtained from the simulations with PRIMO default values agreed with the benchmark data within 2.4% for Clinac 2100. For TrueBeam, the agreement in these parameters was within 1.3%.

Higher differences (up to 2.8% for Clinac 2100, and up to 3.3% for TrueBeam) were found in SBRT– and IMRT–style OF. The limited number of TrueBeam linacs included in the IROC–H database could contribute somewhat to the higher discrepancies found for this linac model. However, it is unlikely that increasing the number of linacs may produce a variation of 2%–3% in the mean value of the experimental OF. That would suggest a remarkable inter–machine variation, which is not observed in the rest of dosimetric parameters. In any case, if more experimental data are available in the future, the results of the present work could be reevaluated.

We investigated three other possible causes that might explain the discrepancies: the application of OF correction factors to IROC–H data, the influence on the estimated dose distributions of the radiation transport approximations introduced in DPM, and the lack of correction in PRIMO of the radiation backscattered from the secondary collimators to the monitor chamber.

### OF correction factors

As commented above, for small fields the ratio of readings from a ionization chamber needs to be corrected to obtain a true dose ratio. IROC–H obtained the small–field OF data with a Exradin A16 microchamber, for which no well–established OF correction factors were available at the time the report was published. That may explain why the IROC–H OF data are uncorrected.

We investigated if applying the TRS–483 [19] OF correction factors for this chamber has an effect on the OF values reported by IROC–H. According to the Table 26 of the TRS–483, for the fields sizes measured by IROC–H a correction factor is needed for the Exradin A16 chamber only for the 2×2 cm^2^ field (with a value of 1.003). For field sizes equal or greater than 3×3 cm^2^, the correction factor is unity. The effect of such a correction for the 2×2 cm^2^ fields is lower than the experimental uncertainties, so it cannot explain the discrepancies observed between IROC–H data and PRIMO simulations.

### DPM vs. PENELOPE

All the previous simulations were run using the DPM algorithm. The rationale was to check the accuracy of the fast algorithm that would be also used to simulate clinical plans. We assessed the difference in simulation efficiency between DPM and PENELOPE with four VMAT plans of common treatment sites: gynecological (2 full arcs), head and neck (2 full arcs), lung (2 half–arcs), and prostate (1 full arc). With the same simulation parameters, and using the same number of computing cores, DPM was about 7 times faster than PENELOPE. The performance gain of DPM comes from simplifications in the particle transport algorithm, and also in the physics models involved [6]. To discard that such simplifications were the cause of the differences with the IROC–H data, we rerun the simulations of the IMRT– and SBRT–style fields with the PENEASY/PENELOPE engine. The results for the Clinac 2100 linac are shown in Fig. 5. The maximum difference between DPM and PENELOPE was 0.7% (*k*=2), well within the statistical uncertainty attained (1.8%, *k*=2). The maximum differences for TrueBeam were smaller than 0.3% (not shown). Hence, at the level of uncertainty attained, the OF results obtained with DPM and PENELOPE are statistically compatible.

### Lack of correction for backscatter radiation into the monitor chamber

The signal from the linac monitor chamber that controls the beam output may be affected by the position of the secondary collimators (jaws), depending on the design of the linac head [1]. In small fields, more radiation backscattered from the jaws will reach the monitor chamber than in large fields. This will cause the linac output to decrease as the field size decreases. This output decrease is included in output factor measurements. However, in Monte Carlo simulations the effect must be accounted for explicitly.

The current method implemented in PRIMO to convert from eV/(g history) to Gy/MU does not correct for variations with the field size of the backscattered radiation into the monitor chamber [20]. However, PRIMO doses could be corrected using the monitor backscatter factor (MBSF) described by Zavgorodni et al. [21]. In that work, the authors obtained experimentally the MBSF for 6 MV beams from Varian 21EX and TrueBeam linacs, for a range of field sizes. For the 6 MV beam from the 21EX linac, they found an MSBF of 0.996 for a 2×2 cm^2^ field size, and of 0.997 for a 3×3 cm^2^ field size (taking as reference the 10×10 cm^2^ field size). For the 6 MV beam from the TrueBeam, the influence of backscatter was even smaller. For small fields with Y jaws above 1 cm, the MSBF can be assumed as unity. Zavgorodni et al. concluded that these values would likely be valid also for 6 MV FFF beams, as previous works had reported very similar backscatter radiation for both beam modalities.

From these results, it is clear that the backscatter correction, although not accounted for by PRIMO, is too small to explain the discrepancies observed in our work between the IROC-H data and the simulation results.

### Final remarks

Although a fine–tuning is possible with PRIMO to closely match simulation results with a particular linac, the results obtained with PRIMO default parameters and DPM algorithm for the Clinac 2100 and TrueBeam linacs are highly consistent with the values reported by IROC–H, with mean differences in absolute value of 1.3% and 0.9%, respectively. Such differences are below the criterion most often used in the radiation oncology community of 2%–3% agreement between the dose calculation of the TPS, and the redundant calculation from an independent software [22].

The parameters included in the IROC–H database used in this work involve only static fields. To confirm the accuracy of PRIMO as an independent calculation system for IMRT/VMAT clinical plans, dosimetric tests on dynamic fields and on MLC characteristics would be also necessary. Such validation for dynamic fields would be a natural extension of the present work.

## Conclusions

The PRIMO default initial beam parameters for 6 MV photon beams from Varian Clinac 2100 linacs and 6 MV FFF photon beams from Varian TrueBeam linacs allow obtaining dose distributions in a water phantom which agree within 3.3% with a database of dosimetric data based on measurements on large series of linacs of the same models. The findings of this work represent a first step in the validation of PRIMO to be used as an independent verification software of radiotherapy plans computed by a treatment planning system.

## Additional file

Additional file 1 Excel file, with additional tables, and images on the EBT3 film experimental setup. (XLSX 470 kB)

## Abbreviations

AAPM: American Association of Physicists in Medicine
CT: computed tomography
DPM: Dose Planning Method
FFF: flattening–filter free
FWHM: full width at half–maximum
IMRT: intensity–modulated radiation therapy
IROC-H: Imaging and Radiation Oncology Core–Houston
MLC: multileaf collimator
MBSF: monitor backscatter factor
OF: output factor
PDD: percentage depth–dose
PSF: phase–space file
SBRT: stereotactic body radiation therapy
TPS: treatment planning system
VMAT: volumetric modulated arc therapy
